# A Report of Haemophilus influenzae Bacteremia With Acute Pelvic Inflammatory Disease

**DOI:** 10.7759/cureus.28970

**Published:** 2022-09-09

**Authors:** Daphne-Dominique H Villanueva, Jordan P Staton, Asmita A Gupte

**Affiliations:** 1 Division of Infectious Diseases, West Virginia University School of Medicine, Morgantown, USA; 2 Obstetrics and Gynecology, North Florida/South Georgia Veterans Affairs (VA) Medical Center, Gainesville, USA; 3 Medicine/Infectious Diseases, North Florida/South Georgia Veterans Affairs (VA) Medical Center, Gainesville, USA; 4 Division of Infectious Diseases and Global Medicine, University of Florida College of Medicine, Gainesville, USA

**Keywords:** bacteremia, bacterial vaginosis, sexually transmitted disease, pelvic inflammatory disease, hemophilus influenzae

## Abstract

Sexually-transmitted organisms that frequently originate from the flora of the lower genital tract are often implicated in pelvic inflammatory disease (PID). *Haemophilus influenzae*, a pathogen found primarily in the upper respiratory tract, has been rarely associated with PID. Here we report a case of a young woman with PID whose blood cultures grew *H. influenzae* biotype II, a reminder that the endometrium can be the source of systemic *H. influenzae* infection when no typical primary focus is found.

## Introduction

In the United States, an estimated 2.5 million women aged 18 to 44 years report a lifetime history of pelvic inflammatory disease (PID) [1]. Many women have minimal symptoms that can go unrecognized. Given the subtle presentation of PID and its impact on reproduction including the risk for ectopic pregnancy and infertility [2], clinicians should maintain a low threshold for its diagnosis. Immediate antimicrobial therapy for typical organisms known to cause PID, including *Neisseria gonorrhoeae* and *Chlamydia trachomatis*, is essential in treatment. In order to avoid serious complications, it is common for emergency care providers to start therapy for these most common pathogens empirically. This case highlights the importance of identifying the source and species of infection in order to provide appropriate therapy.

## Case presentation

A healthy 35-year-old female veteran with a history of uterine fibroids and chronic pelvic pain presented to the emergency department (ED) with a three-day history of lower abdominal pain and one episode of a 100.3 ⁰F fever. She was sexually active with one male partner and was not on contraception due to a desire to become pregnant. She reported a remote history of chlamydia and a more recent history of treatment for bacterial vaginosis (BV). The patient had no recurrence of abnormal vaginal discharge after treatment of BV and she had no diarrhea or dysuria to suggest a gastrointestinal (GI) or urinary source of her pain.

On examination, she was hemodynamically stable with a blood pressure of 119/84 mmHg and a heart rate of 85 beats per minute. She had lower abdominal tenderness to deep palpation. A pelvic exam revealed thin white vaginal discharge with otherwise normal mucosa, a mildly enlarged uterus with irregular borders and tenderness to palpation over the fundus and a palpable right lateral fibroid, no adnexal masses or fullness but mild tenderness to palpation in the right lower quadrant. She had no flank tenderness and the rest of the physical exam was unremarkable.

**Figure 1:**
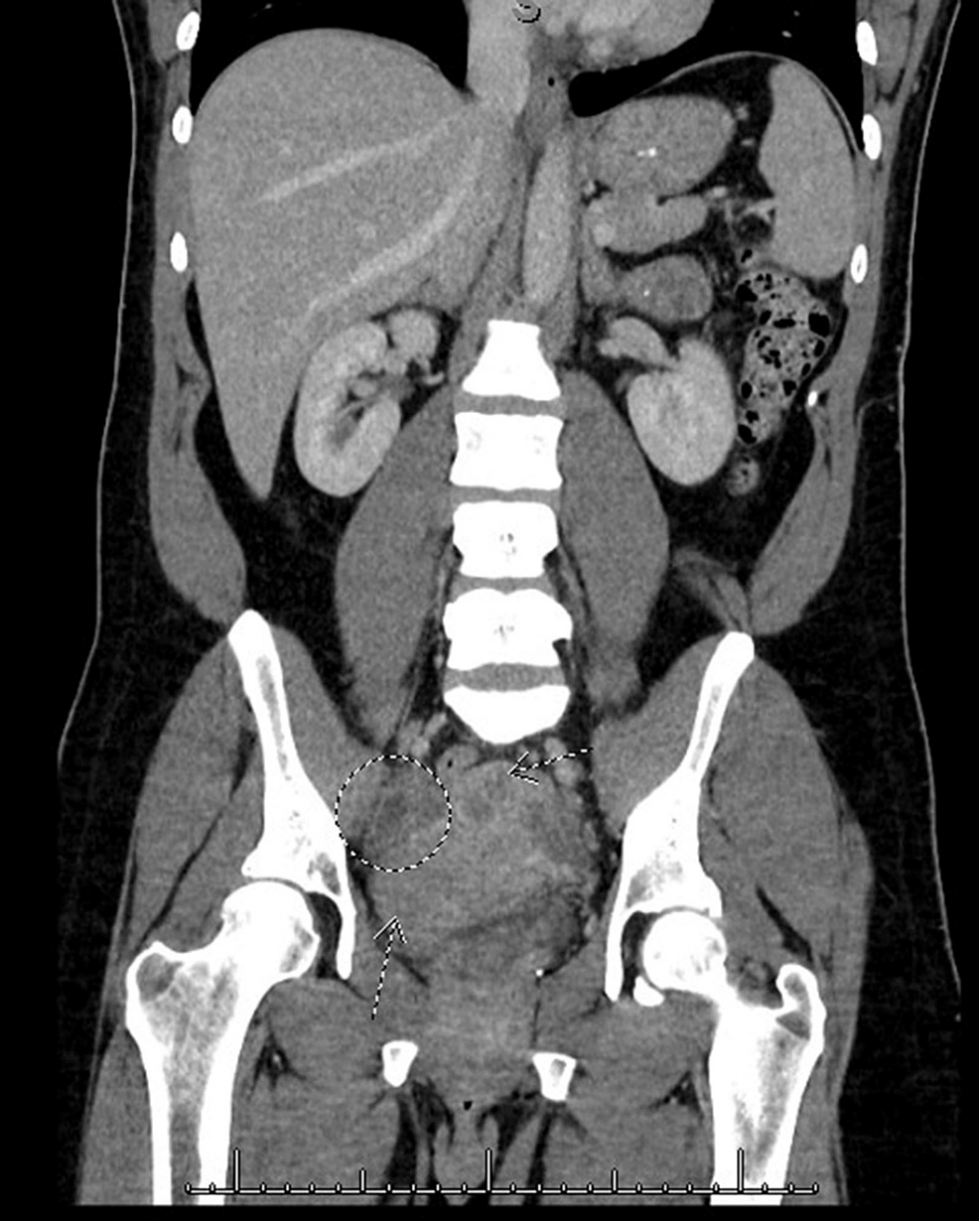
Computed tomography scan of the abdomen Computed tomography of the abdomen and pelvis demonstrating the right ovary with cystic changes (circle) and uterine fibroids (arrows).

**Figure 2:**
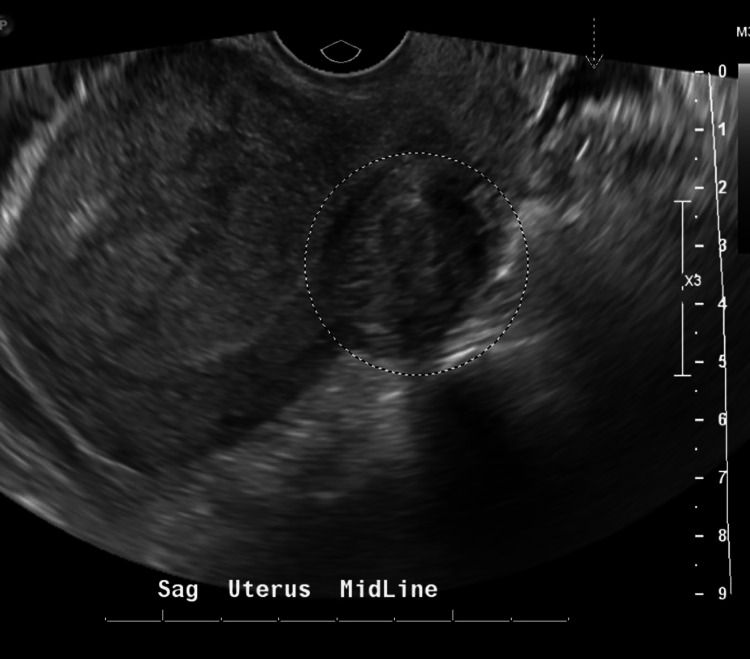
Transvaginal pelvic ultrasound image Transvaginal pelvic ultrasound showing fibroids (circle) with only trace free fluid in the pelvis (arrow).

Initial laboratory examination revealed leukocytosis of 14.01 k/mm^3 ^(reference range 4.6 - 10.8 k/mm^3^), a negative urine pregnancy test, and a normal urinalysis. Blood and urine cultures were obtained. A computed tomography (CT) scan of the abdomen and pelvis demonstrated uterine fibroids and a 2.1 x 1.6 cm area of cystic change in the right adnexa that likely represents normal findings for the patient's age (Figure 1). There were no extra-pelvic findings of concern. A transvaginal pelvic ultrasound confirmed two 3-4 cm subserosal fibroids (unchanged from previous imaging) and normal ovaries, with only trace free fluid in the pelvis and no tubo-ovarian abscess (Figure 2). A urine polymerase chain reaction (PCR) test was negative for *C. trachomatis* and *N. gonorrhoeae* but a vaginal BD Affirm^TM^ (Becton, Dickinson and Company, NJ) wet prep swab was positive for *Gardnerella vaginalis*.

The initial working diagnosis was PID in a patient with recurrent BV. She was given one dose of intramuscular ceftriaxone 250 mg in the ED and was sent home on oral doxycycline 100 mg twice a day and clindamycin 300 mg three times a day. However, she was called back to the ED after blood cultures from her initial visit came back positive with gram-negative rods (GNR). See Table 1 for a summary of the patient’s laboratory results upon return to ED compared with her test results on initial presentation.

**Table 1:** Laboratory results on initial presentation and upon return to Emergency Department (ED)

Laboratory test	Results on initial presentation	Results upon return to ED	Reference range
White blood cell count	14.01 k/mm^3^	7.69 k/mm^3^	4.6 - 10.8 k/mm^3^
Hemoglobin	11.7 g/dL	12.0 g/dL	13.9 - 18 g/dL
Hematocrit	34.5%	35.6%	41 - 52%
Platelet count	310 k/mm^3^	275 k/mm^3^	130 - 440 k/mm^3^
Creatinine	0.9 mg/dL	0.8 mg/dL	0.5 - 1.2 mg/dL
Aspartate aminotransferase (AST)	13 U/L	15 U/L	0 - 45 U/L
Alanine aminotransferase (ALT)	13 U/L	12 U/L	0 - 40 U/L

In the interim, she complained of worsening abdominal pain and new-onset diarrhea. At the time of admission, the most unifying diagnosis was PID although a GI source could not be ruled out as the patient now had diarrhea. GI work-up including stool culture and *Clostridioides difficile* PCR were negative. The GNR in her blood cultures then speciated into *Haemophilus influenzae*, which was found to be beta-lactamase positive by rapid disk test. Upon further questioning, the patient reported chronic non-productive cough which she attributed to cigarette smoking. Further investigation into potential respiratory sources of the bacteremia was negative, including a chest X-ray that showed no acute cardiopulmonary disease and a sputum culture that was collected two days into hospitalization that yielded normal upper respiratory flora. At this point, it was assumed that the source of her *H. influenzae*bacteremia was the uterus, where this organism has been known to cause acute endometritis in rare cases.

Immediate antimicrobial therapy with intravenous cefepime 2 g every 12 hours was started for GNR bacteremia and the patient was continued on oral doxycycline 100 mg twice a day and oral metronidazole 500 mg three times a day for PID with BV. Once the GNR was identified as *H. influenzae*, the intravenous antibiotic was transitioned to oral levofloxacin 750 mg once daily and the oral doxycycline and metronidazole were continued for a total of 14 days.

The blood *H. influenzae* isolate was sent to the state laboratory for typing and was found to be nontypable biotype II by gene capsule analysis. Her acute pain symptoms resolved after appropriate antimicrobial treatment, and she did not develop a more concerning systemic response to her bacteremia. The patient was offered the full range of medical and surgical treatment options for her symptomatic uterine fibroids but declined any hormone-based therapy due to the prioritization of her fertility goals. She planned to see her gynecologist as an outpatient to consider elective myomectomy after the resolution of her acute illness but was unfortunately lost to follow-up. It is noted that she has visited the ED on at least two subsequent occasions with complaints similar to those within this case, and has been treated empirically for recurrent PID.

## Discussion

PID results in a direct spread of microorganisms from the vagina or endocervix to the endometrium and fallopian tube mucosa [3]. Empiric antibiotic regimens provide broad-spectrum coverage of pathogens that frequently originate from the flora of the lower genital tract including *N. gonorrhoeae*, *C. trachomatis*, anaerobes, gram-negative bacteria, and streptococci [4]. Uncommonly, respiratory pathogens including *H. influenzae* have also been isolated from the upper genital tracts of women with symptomatic PID suggesting that these bacteria ascend from the vaginal canal [5,6].

*H. influenzae* is a small, nonmotile gram-negative coccobacillus that is a human pathogen found principally in the upper respiratory tract. *H. influenzae* capsular strains have six serotypes designated a to f based on antigenically distinct capsular polysaccharide types. Strains of *H. influenzae *that lack a polysaccharide capsule demonstrate substantial genetic diversity and are generally referred to as nontypeable [7]. Nontypeable* H. influenzae* is a common cause of otitis media in children and exacerbation of chronic obstructive pulmonary disease in adults. The widespread use of *H. influenzae* type b (Hib) conjugate vaccine was accompanied by a shift in invasive *H. influenzae *disease, with nontypeable strains replacing capsular type b strains as the most common bloodstream isolate [8]. Systemic infection with nontypeable* H. influenzae* often occurs in neonates or in older persons with underlying comorbidities such as cardiopulmonary disease or cancer [9]. When nontypeable *H. influenzae *causes bacteremia, the respiratory tract is the usual source of infection. Rarely, nontypeable *H. influenzae *can also cause acute endometritis, which is mostly reported in the postpartum setting or associated with an intrauterine device (IUD). This case is unusual in that neither of these clinical situations was present.

One similar case report identified a non-IUD-associated *H. influenzae* endometritis in a 48-year-old woman where PCR identified nontypeable *H. influenzae* on the endometrial tissue biopsy [5]. This study did not identify the biotype of the strain that was isolated. In a series of 114 *H. influenzae* and *H. parainfluenzae *isolates from various genital, mother-infant, and neonatal infections, strains were characterized according to biotype and serotype. In this series, 62% of PID cases were related to the presence of an IUD. *H. influenzae* biotype I was the most frequent isolate in PID whereas biotype IV prevailed in postpartum endometritis [6]. In our case, biotype II was isolated in the bloodstream.

Although an endometrial biopsy was not performed on our patient, the diagnosis of acute PID was supported by the clinical presentation with lower abdominal pain, tenderness on palpation during a pelvic examination, and imaging showing free pelvic fluid. Certainly, this infection might have been attributed solely to BV if not for the positive blood cultures. It could be said that a source of *H. influenza* was not confirmed given the lack of definitive evidence which could have been provided by an endometrial biopsy specimen. However, she responded to appropriate antimicrobial therapy and therefore an invasive procedure was not considered to be warranted to guide her treatment. In the absence of other localizing symptoms, a clear chest X-ray, and normal sputum cultures, these findings suggest that the endometrial infection is the primary source of the patient’s *H. influenzae *bacteremia. The reason for her genital *H. influenza* infection and subsequent bacteremia remains unclear but one could argue that testing her sexual partner’s oropharynx could potentially relate her bacteremia to oral-genital sexual contact.

It is worth noting also that the patient had concurrent BV which is common in women with confirmed PID [10]. Although the pathogenesis of BV remains unclear, it is thought to represent an imbalance of the vaginal microbiota from the predominance of lactobacilli to a higher concentration of other organisms involving not only *G. vaginalis* [11]. Recurrent BV infections are known to increase the risk of co-infection with other urogenital pathogens.

## Conclusions

The etiology of PID depends on careful evaluation of the history, physical examination, and laboratory tests. Lack of improvement of symptoms on empiric antimicrobial therapy should prompt further investigation with blood cultures, testing the patient’s sexual partner, and ideally obtaining tissue samples for pathologic diagnosis and molecular testing. Through this case, we hope to raise awareness regarding pathogens from the respiratory tract that in rare cases cause infections in the upper genital tracts of women.
